# Acute obstructive pyelonephritis due to pelvic organ prolapse: a case-based review of the literature

**DOI:** 10.2144/fsoa-2020-0204

**Published:** 2021-03-11

**Authors:** Georges Abi Tayeh, Eddy Lilly, Melissa Abi Antoun, Rhea Akl, Georges Mjaess, David Atallah, Maroun Moukarzel

**Affiliations:** 1 Department of Urology, Hotel-Dieu de France, University of Saint Joseph, Beirut, Lebanon; 2 Department of Obstetrics & Gynaecology, Hotel-Dieu de France, University of Saint Joseph, Beirut, Lebanon; 3 Department of Radiology, Hotel-Dieu de France, University of Saint Joseph, Beirut, Lebanon

**Keywords:** hydronephrosis, pelvic organ prolapse, pyelonephritis, pyonephrosis

## Abstract

Pelvic organ prolapse (POP) can lead to acute bilateral obstructive pyelonephritis (ABOP) due to bilateral ureteral compression. When this occurs, conservative treatment through POP reduction, intravenous antibiotics and supportive care seems to provide an interesting option in the wait of definitive management of POP. The cornerstone of ABOP management, which is the emergent urinary drainage, seems to have many drawbacks in this context due to both technical and patient-related criteria, making it invasive and compromising patient safety and comfort in many settings. Here, we review the management of ABOP and provide a case of an acute obstructive pyelonephritis due to POP.

## Lay abstract

Pelvic organ prolapse (POP) is a common condition arising in a considerable number of women. While its main repercussions include pelvic pain or discomfort, disruption of sexuality and quality of life, pelvic organ prolapse may also lead to life-threatening conditions, namely acute obstructive pyelonephritis (AOP). We hereby expose the case of an AOP due to POP while performing a review of the literature on the proposed management and how it differs from the classic management of AOP.

Pelvic organ prolapse (POP) is a common condition that can have severe repercussions on women’s daily activities, sexuality and body image [1]. It is difficult to determine the prevalence of POP because of various classification schemes and the ongoing dilemma of whether to include or not asymptomatic women in prevalence studies [2]. In fact, it is estimated that around 3–6% of women would eventually report symptoms attributed to POP while roughly 50% of women are diagnosed with POP when prevalence is measured based on pelvic examination [3].

Symptomatic women mostly report vaginal bulging. Concomitant pelvic symptoms that are also reported mainly include urinary urgency, frequency, urinary incontinence, voiding dysfunction and fecal incontinence [4].

When left untreated, POP can lead to recurrent urinary tract infections, hydronephrosis, pyonephrosis and even end-stage renal disease [5].

## Case presentation

We herein report the case of a 76-year old menopaused multiparous female with a history of three normal vaginal deliveries, well-controlled hypertension and Type 2 diabetes mellitus, who presented to the emergency department for acute obstructive bilateral pyelonephritis having complained of sustained high-grade fever, left flank pain and severe acute storage low urinary tract symptoms. Physical exam revealed a high-grade fever with a grade 4 anterior and apical compartment prolapse with no urine leakage on prolapse reduction testing, and bilateral costovertebral angle tenderness.

The patient had undergone a laparoscopic sacrohysteropexy in early 2018 as a cure for her grade 4 debilitating anterior and apical compartment prolapse, also accountable for a bilateral hydronephrosis at the time. She started complaining a year later from a clinical relapse of her cystocele manifesting as a complete vaginal eversion and intermittent self-limiting macrohematuria.

Her past surgical history also includes a left quadrantectomy and axillary lymph node dissection for a luminal A left breast cancer in 2008, followed by adjuvant radiotherapy, chemotherapy according to an AC-T regimen: 4 cycles of combined doxorubicin and cyclophosphamide followed by 4 cycles of paclitaxel (Taxol) and Tamoxifen-based hormonotherapy.

Furthermore, she was treated for recurrent upper and lower urinary tract infections (UTIs). The last infectious episode occurred in January 2020 when she was treated for a community acquired nonobstructive pyelonephritis, incriminating a penicillin-resistant *Escherichia coli*. A renal and pelvic ultrasound performed in the aforementioned context showed no hydronephrosis and a 170 ml postvoid residue. A therapeutic trial by once daily tamsulosin was initiated in an attempt to improve voiding as a mean to reduce the frequency of UTIs. The patient was also instructed to perform clean intermittent catheterization of the bladder once daily but was never compliant.

**Figure 1.**
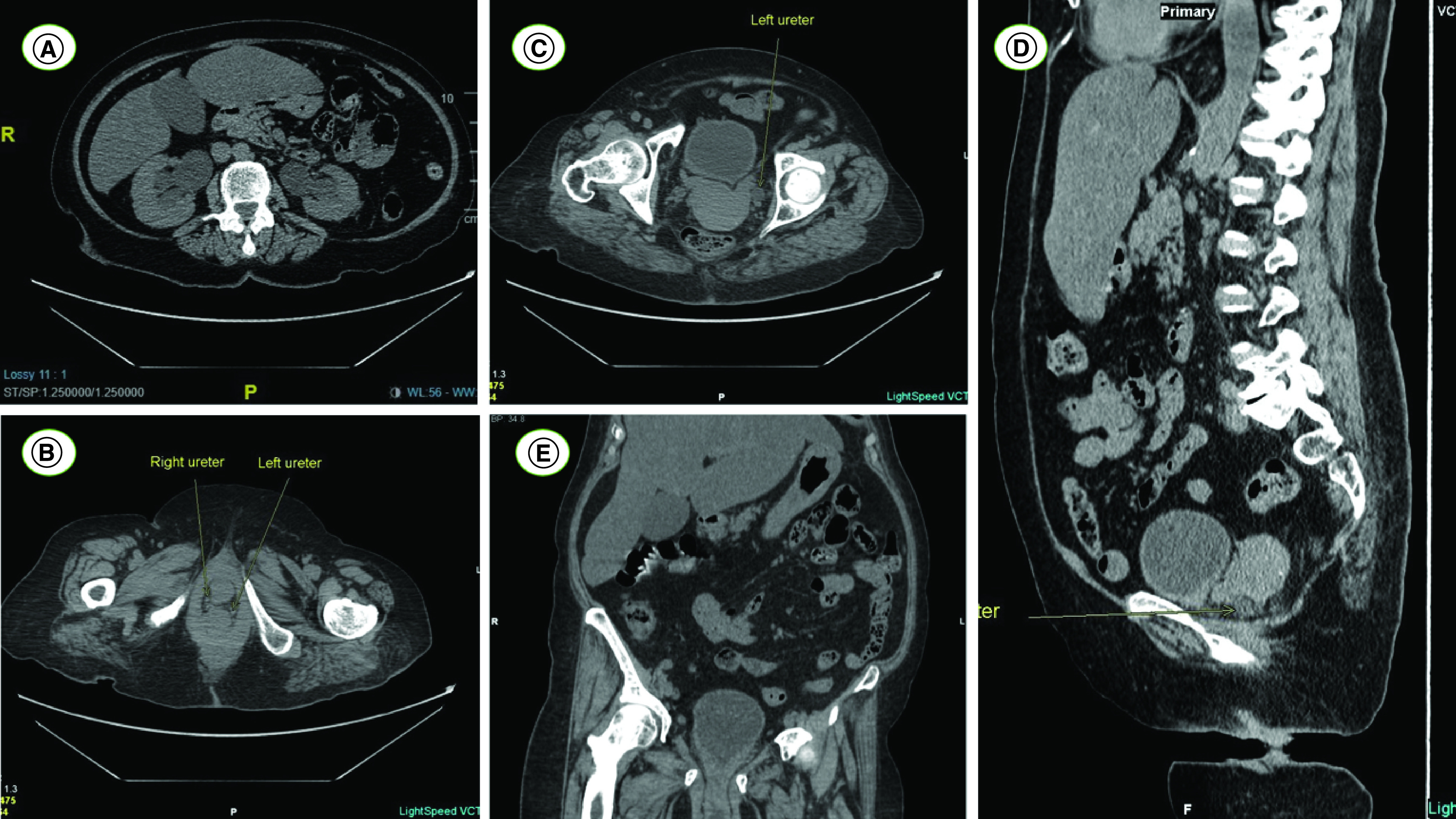
Computed tomography scan of our patient. **(A)** Computed tomography scan without intravenous contrast showing severe bilateral hydronephrosis; **(B)** CT scan without intravenous contrast showing bilateral ureteral compression by the urogenital hiatus; **(C)** CT scan without intravenous contrast showing bilateral hydroureters; **(D)** CT scan without intravenous contrast showing left ureter compression between uterine fundus and pelvic diaphragm; **(E)** CT scan without intravenous contrast showing grade 4 cystocele and associated colpocele.

Abdominal and pelvic computed tomography (CT) scan without injection of intravenous contrast showed severe bilateral hydronephrosis (Figure 1A), associated with bilateral hydroureters extending to the urogenital hiatus (Figure 1B & C), where both of the ureters seem to be directly compressed between the uterine fundus and the pelvic diaphragm (Figure 1D) due to POP (Stage 4 Ba prolapse and associated Stage 2 C prolapse according to POP-Q classification) (Figure 1E). Diffuse thickening of the bladder wall could be seen as well, with no evidence of bladder overdistension. CT imaging was not in favor of the eventuality of a ureteral stone migration and revealed no peri-renal fat stranding, nor indirect signs of inflammation.

Blood work revealed a white blood cell count of 8.0 × 10^3^/dl, a mild anemia, a creatinine level of 0.81 mg/dl, C-reactive protein (CRP) level was 24.6 mg/l. Urinalysis showed combined hematuria and pyuria with urine culture demonstrating the growth of >10∧6 *Klebsiella pneumoniae* with extended-spectrum betalactames secretion. The patient was treated empirically then according to bacterial sensitivity with intravenous piperacillin-tazobactam (4.5 g intravenously perfused over 6 h, four-times daily). The patient was reluctant to undergo a bilateral emergent nephrostomy and thus 1 g of amikacin was administered once daily, intravenously, for 2 consecutive days due to the sustained high-grade fever with a preserved stable hemodynamic state. Vaginal pessary insertion was proposed but the patient could not find the prescribed device and, therefore, the POP was only reduced manually in a temporary fashion.

For the current context, a specialized infectious diseases consultation was sought and piperacillin-tazobactam was started empirically then adjusted according to culture. The patient was apyretic on day 2 of her admission. Due to the rapid recurrence of the prolapse, in the year following her laparoscopic sacrohysteropexy, and due to the patient’s preference, a shared decision was made to perform a transvaginal cure of the prolapse on day 3 of her admission. The surgery consisted of a vaginal hysterectomy, with cystocele repair through an anterior colporrhaphy (consisting of a suture-plication of the vesico-vaginal Halban's fascia – using nonabsorbable multifilament braided separate sutures) associated with Richter’s sacrospinous ligament fixation of the prolapsed vaginal vault using a vaginal flap for prevention of a subsequent vaginal vault prolapse.

Vaginal packing was left overnight, and the Foley bladder catheter was removed 48 h later. PVR was 300 ml, measured by ultrasound testing and the patient left with instructions to perform clean intermittent catheterization twice daily and ertapenem intramuscular injections for a total antibiotic treatment of 14 days.

The patient had no PVR on short term follow-up but manifested a new-onset stress urinary incontinence (SUI) and is posted for urodynamic evaluation.

## Review

Hydronephrosis in POP is not a rare condition and must be screened for, in the context of a patient presenting with POP: in fact, a systematic review carried out by Siddique *et al.* reported that the prevalence of hydronephrosis in POP ranges from 3.6 to 30.6% depending on the studies and showed that uterovaginal prolapse was more accountable for hydronephrosis and especially severe hydronephrosis than vaginal vault prolapse [6]. No study demonstrated a statistically significant association between severity or duration of the prolapse and stage of the hydronephrosis, but a more severe prolapse was associated with a higher prevalence of hydronephrosis. Nonetheless, POP severity was not found to cause higher serum creatinine levels [6]. Consequently, creatinine levels were within normal values and remained as such regardless of the chronicity and severity of POP. Also, it has been suggested that creatinine levels do not predict the occurrence of hydronephrosis nor its severity in the context of POP [7]. The causality link between POP and ureterohydronephrosis is strengthened by the reversible aspect of the latter once the prolapse has been surgically cured, preventing future complications [8].

The precise mechanism of hydronephrosis in the exposed case is a direct compression of ureters by the genital hiatus against the uterus fundus and the pelvic diaphragm leading to bilateral hydronephrosis. Another suggested mechanism hypothesized that the cardinal ligaments, laterally attached to the fascia above the pelvic sidewall and medially to the cervix and corpus uterus, form a loop over the ureter and force it down as the uterus descends, thereby forming the ureter kink [9].

Treatment of hydronephrosis attributed to POP should be oriented toward a strategy that prevents complications involving the upper urinary tract, mainly resolving the hydronephrosis; while still focusing on the patient-oriented symptomatic aspect of the condition [10].

In the presented case, the patient refused emergent bilateral percutaneous nephrostomy as a means of urinary diversion in the context of acute obstructive pyelonephritis. However, after achieving apyrexia through antibiotics and supportive treatments alone, routine vaginal hysterectomy followed by anterior colporrhaphy were performed as a cure for cystocele and sacrospinous colpopexy was carried out to treat the vaginal vault prolapse. The vaginal approach was chosen bearing in mind a previous laparoscopic sacrohysteropexy. In fact, the surgical reduction could be done either by a suprapubic or vaginal approach, and should preferably include a hysterectomy when possible [10].

Although this surgery increases the risk of SUI, our patient did not complain of incontinence at her initial presentation but had a PVR of 300cc and had to undergo clean intermittent catheterization thrice daily. The onset of urinary retention after POP repair is not uncommon with an incidence of 6–29% reported in the literature, although the exact cause is not fully understood yet [11–14]. Risk factors that were found to be associated with a higher rate of postoperative urinary retention are: old age, especially when undergoing vaginal POP repair [15]; high grade cystocele; severe intra-operative blood loss; application of levator and the Kelly plication technique; postoperative pelvic hematoma; and early postoperative bladder catheter removal [11,12,16]. Nonetheless, once her POP was surgically cured, she managed to fully empty her bladder, while developing a new-onset SUI although tension-free vaginal tape (TVT) maneuver performed pre-operatively was negative.

A retrospective study conducted by Leanza *et al.* managed to make the impression that a vaginal hysterectomy followed by vaginal apex suspension and anterior/posterior colporrhaphy can completely resolve or improve hydronephrosis caused by POP, and they also suggested that a prepubic tension-free incontinence cystocele treatment helps maintain continence [17].

However, when hydronephrosis due to POP, notably a high-grade cystocele is complicated by acute bilateral obstructive pyelonephritis (ABOP), the context deviates from a deferred emergency aiming to prevent renal function deterioration to a life-threatening condition requiring imminent medical attention. One must bear in mind that endoscopic retrograde drainage is somehow technically challenging in high-grade cystoceles and that percutaneous drainage is a less attractive option when patient comfort is taken into consideration.

This is the reason why many cases have addressed this dilemma, trying to establish a safe balance between patient comfort and safety. Cases of ABOP due to ureteral compression by a POP are scarce and management varied from one case to another. Some authors opted for a trial of urinary drainage to relieve obstruction. As a matter of fact, the literature reports a case of a patient with pyonephrosis and end-stage renal disease managed by bilateral ureteral double J stent insertion under local anesthesia due to the patient-related contraindication to anesthesia [11–14]. Another case is that of an 80-year-old woman who presented with a life-threatening POP with obstructive pyelonephritis and disseminated intravascular coagulation (DIC) who was treated with urgent drainage of infected urine by bilateral ureteral stents and pessary insertion, with antibiotics and supportive treatment [18]. Therefore, it seems that urinary drainage still compromises patient safety either because of the septic context upon presentation and/or due to the usually frail elderly presenting with ABOP.

Thus, some patients have been treated conservatively due to patient preference, unavailable treatment modalities or contraindications to surgery or percutaneous urinary drainage.

Ota *et al.* reported a case of bilateral hydronephrosis secondary to uterine prolapse with urinary tract infection treated with intravenous meropenem and vaginal pessary insertion that resolved hydronephrosis as demonstrated by a follow-up CT scan [19]. The same conclusion was deducted by Young *et al.* after managing the case of a patient in septic shock attributed to ABOP traceable to POP; insertion of ring pessary and reduction of the prolapse was also enough to drain the infected urine [20]. As a matter of fact, even the manual reduction of the uterovaginal prolapse is sufficient to provide a temporary but nonetheless efficient drainage of infected urine, as demonstrated by a prompt drainage of urine by a bladder catheter, placed in a bladder that was prevented from filling due to previous ureteral compression [21]. The cornerstone of treatment in most cases, providing the best and safest therapeutic results, is to perform a prolapse reduction.

We consider our described case to be interesting since it describes another rare case where ABOP due to POP was treated without endoscopic or percutaneous urinary drainage and our patient still improved clinically and biologically relying on exclusive antibiotic therapy. Vaginal pessary insertion was proposed but the patient could not find the prescribed device and therefore the POP was only reduced manually in a temporary fashion.

In the absence of comparative studies due to the rarity of reported cases, it seems that conservative management of ABOP in the context of POP with intravenous antibiotics, temporary reduction of POP through pessaries or manually if possible and adequate supportive treatment may lead to a favorable course of disease until a radical suspensory management is carried out, preferably in the same hospital stay.

This is further supported by the fact that hydronephrosis in the setting of POP rarely leads to a compromise of renal function as mentioned above, suggesting an indolent course of the hydronephrosis once supportive management can be proposed.

## Conclusion

POP can lead to hydronephrosis that can cause UTI and end-stage renal disease if left untreated [5]. Patients presenting with POP should be screened for hydronephrosis using renal ultrasound or CT scan, since creatinine level does not predict hydronephrosis nor its severity [7]. When ABOP occurs due to ureteral compression by POP, conservative treatment through POP reduction, intravenous antibiotics and supportive care seems to provide an interesting option in lieu of a definitive management of POP. Emergent urinary drainage, the cornerstone of ABOP management, seems to have many setbacks in this context due to both technical and patient-related criteria, making it somehow invasive and compromising patient safety and comfort in many settings.

## Future perspective

Despite the global consensus on the necessity of emergent urinary drainage in the setting of ABOP due to POP, research must mainly focus on determining the patients at risk of future complications, namely infections or kidney damage, inciting prompt surgical definitive interventions. These risk factors may include clinical, patient-related, biological and imaging elements. Furthermore, effort should be made toward finding the best definition of ABOP in this particular context in order to be able to provide a better prevalence assessment and consequently unified diagnostic and management guidelines.

**Executive summary**

- Pelvic organ prolapse (POP) is a common condition that can have severe repercussions on women’s daily activities, sexuality and body image.
- When left untreated POP can lead to recurrent urinary tract infections, hydronephrosis, pyonephrosis and even end-stage renal disease.

**Case presentation**

- We present the case of a 76-year old menopaused multiparous female who presented to the emergency department for acute obstructive bilateral pyelonephritis due to a grade 4 anterior and apical compartment prolapse.
- Computed tomography scan without injection of intravenous showed severe bilateral uretero-hydronephrosis with both ureters compressed between the uterine fundus and the pelvic diaphragm.
- The patient refused bilateral nephrostomy and was treated by intravenous piperacillin-tazobactam and manual reduction of the cystocele as vaginal pessaries were not available.
- The patient improved clinically and biologically and ultimately underwent a transvaginal cure of the prolapse on day 3 of her admission.

**Discussion**

- The prevalence of hydronephrosis in POP ranges from 3.6 to 30.6%.
- The precise mechanism of hydronephrosis in the exposed case is a direct compression of ureters by the genital hiatus against the uterus fundus and the pelvic diaphragm.
- The best approach in an emergency setting would include intravenous antibiotics and cystocele reduction, maintained by vaginal pessaries.
- Definitive treatment includes a surgical cure of the POP.
- Future research work must focus on establishing formal guidelines for management of similar cases and more importantly on elucidating clinical, biological and imaging predictors of acute obstructive pyelonephritis in patient with hydronephrosis due to POP.
